# Low-Cost Electromagnetic Split-Ring Resonator Sensor System for the Petroleum Industry

**DOI:** 10.3390/s22093345

**Published:** 2022-04-27

**Authors:** Alejandro Rivera-Lavado, Alejandro García-Lampérez, María-Estrella Jara-Galán, Emilio Gallo-Valverde, Paula Sanz, Daniel Segovia-Vargas

**Affiliations:** 1 Signal Theory and Communications Department, Carlos III University of Madrid, 28903 Madrid, Spain; arivera@ing.uc3m.es (A.R.-L.); alamperez@tsc.uc3m.es (A.G.-L.); 2 Yebes Observatory, Dirección General del Instituto Geográfico Nacional, 19141 Yebes, Spain; 3 Indra Sistemas S.A., 28108 Madrid, Spain; mejara@indra.es (M.-E.J.-G.); egallo@minsait.com (E.G.-V.); 4 REPSOL S.A., 28045 Madrid, Spain; psanzs@repsol.com

**Keywords:** effective permittivity, resonator, sensor, split-ring resonator (SRR), submersible sensor

## Abstract

The use of a low-cost split-ring resonator (SRR) passive sensor for the real-time permittivity characterization of hydrocarbon fluids is proposed in this paper. The characterization of the sensor is performed through both full-wave simulation and measurements. Thanks to the analysis of several crude samples, the possibility of discrimination between different types of crude and the estimation of several of their properties are demonstrated. Between them, the estimation of sulfur, aromatic hydrocarbons, and salt-water concentrations either in normal ambient conditions or in a high-pressure and high-temperature environment can be mentioned. Experiments were run both at normal ambient conditions and pressures up to 970 bar and temperatures up to 200 °C.

## 1. Introduction

Intelligent well technology allows an efficient operation for both the oil and gas industry. The use of downhole sensors has become popular since it allows for the continuous and real-time monitoring of relevant reservoir factors, such as flow and pressure control, sand and water monitoring [1,2], and leakage detection [3], among others. Gathered data can be processed, analyzed, and used for closed-loop control, well management, and decision making in the extraction, transportation, and processing activities. Moreover, measuring the refractive index (or the permittivity) of the extracted fluid allows for the estimation of other relevant thermodynamic and physical properties, such as critical constants, and average molecular weight, density, viscosity, thermal conductivity, and boiling point [4].

The development of suitable downhole sensors is challenging since a well’s depth can be between 1000 m and 4000 m [2]. These sensors must stand in a high-pressure (above 900 bar) and high-temperature (above 150 °C) environment. This makes it hard to obtain durable and reliable active sensors. Passive devices are more robust but suffer from long-distance communication issues, such as high losses and thermal noise.

Until now, available sensors mostly rely on optical [5,6,7,8], acoustic [9,10,11,12], and radio frequency (RF) [13,14] technologies. RF sensors are specially attractive for downhole applications, since they are easy to manufacture, cost-effective, and robust. Since they can be easily integrated inside well pipes, they are especially suitable for real-time crude properties estimation.

This paper proposes the use of RF split-ring resonators (SRR) as downhole passive sensors for real-time crude monitoring through permittivity estimation. SRR [15] and complementary SRR (CSRR) [16] can be used for measuring a wide variety of magnitudes, such as alignment [17], displacement [18], rotation [19], speed [20], blood glucose [21], and thickness [22,23]. Both SRR [23,24] and complementary SRR [25,26,27] sensors can be used for solids and liquids permittivity characterization. A cost-effective RF sensor interrogator is also introduced in this contribution. It can be placed in the well pad and connected to the downhole sensor trough a low-loss coaxial cable. This device can work as a stand-alone system that can be controlled via standard commands for programmable instruments (SCPI)-compatible commands through a TCP/IP network or can be integrated into a multi-sensor system. In our particular setup, a control system that implements a support vector machine (SVM) classifier combines the measurement of different sensors. Since all the data is normalized before the processing, calibrating the permittivity estimation in the interrogator is not required.

The rest of this manuscript is organized as follows. In Section 2, we introduce our proposed SRR sensor design for downhole crude permittivity estimation. The ability to discriminate between different crude samples and the determination of the sulfur, aromatic hydrocarbons, and salt-water concentrations is demonstrated in Section 3. In Section 4, we introduce an autonomous remotely controlled SRR sensor interrogator that allows real-time crude monitoring. The evaluation of the whole system in a high-pressure and high-temperature setup is shown in Section 5. The interrogator performance is compared in terms of stability and standard deviation with the ones obtained when using a conventional and expensive vector network analyzer (VNA).

## 2. Submersible Split-Ring Resonator-Based Sensor

**Figure 1:**
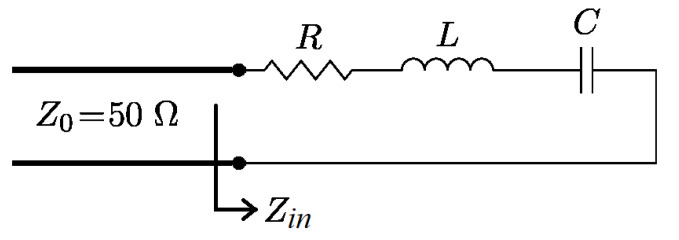
Model of a serial RLC resonator excited by a transmission line.

An SRR resonator excited by an open-ended microstrip line is used for determining the electrical properties of the surrounding fluid. The electric parameter to be measured is the reflection coefficient $\Gamma =S_{11}$. Figure 1 shows the sensor model.

The complex SRR resonator input impedance $Z_{in}\left(\omega \right)$ has a frequency-independent real part, *R*, and an imaginary one, X(ω), which take both positive and negative values at different frequencies. Around the resonant frequency $f_{0}$, the input impedance can be modeled with the following equation

(1) $$Z_{in}\left(\omega \right)=R+j\left(\omega L-\frac{1}{\omega C}\right)=R+jX\left(\omega \right)$$

The quality factor *Q*, the coupling factor *s*, and the resonant frequency $f_{0}$ are given as

(2) $$Q_{0}=\frac{\omega_{0}L}{R}$$

(3) $$s=\frac{Z_{0}}{R}$$

(4) $$f_{0}=\frac{\omega_{0}}{2\pi }=\frac{1}{2\pi \sqrt{LC}}\propto \frac{1}{\sqrt{\varepsilon_{{}_{\mathrm{eff}}}}}$$

$f_{0}$ is determined by the structure dimensions and the effective permittivity $\varepsilon_{\mathrm{eff}}$, which depends on the substrate permittivity and the surrounding fluid permittivity $\varepsilon_{{}_{\mathrm{LUT}}}$. Any increase in the liquid-under-test (LUT) permittivity $\varepsilon_{{}_{\mathrm{LUT}}}$ leads to a reduction in $f_{0}$, as will be shown later in our sensor full-wave simulations. The working frequency, $f_{0}$, has been set in the UHF frequency band in order to reach larger propagation distances and show higher resolution in the variations of fluid surrounding the sensor. However, the use of frequencies in the low microwave band makes the sensor have bigger sizes. In order to reduce the dimensions of the sensor’s high permittivity substrates, Arlon AR1000 with a relative permittivity $\varepsilon_{r}=10$ can be used. For avoiding a low sensitivity due to this relatively high permittivity, a thickness of 1.27 mm is chosen. Substrate losses are relatively low (tan(δ)=0.003) at the working frequencies. Furthermore, according to our tests, this substrate can stand the expected level of pressure and temperature.

**Figure 2:**
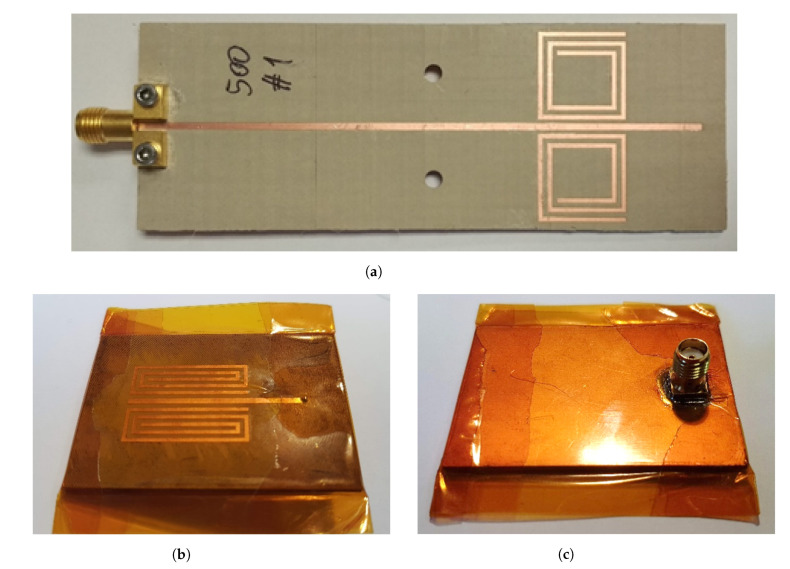
Manufactured A-type (**a**) SRR resonator-based sensor. B-type front (**b**) and back (**c**) view.

Several designs have been tested. In all of them, two SRRs are placed close to an open ending in a symmetric configuration around the microstrip line. Figure 2 shows a picture of the A-type (Figure 2a) and the B-type (Figure 2b,c sensor prototype. Both designs have a resonant frequency in the range of the 500 MHz band ($f_{0,air}=474.5$ MHz for the A-Type and $f_{0,air}=470.22$ MHz for the B-Type). Due to the manufacturing tolerances, each unit may have a different $f_{0,air}$. Such bias can be easily compensated by considering the frequency difference $\Delta f=f_{0}-f_{0,air}$ instead of absolute frequency values for samples characterization. The A-type sensor has a dimension of 95.25×32.22 mm^2^, while the B-type is a more compact version.

**Figure 3:**
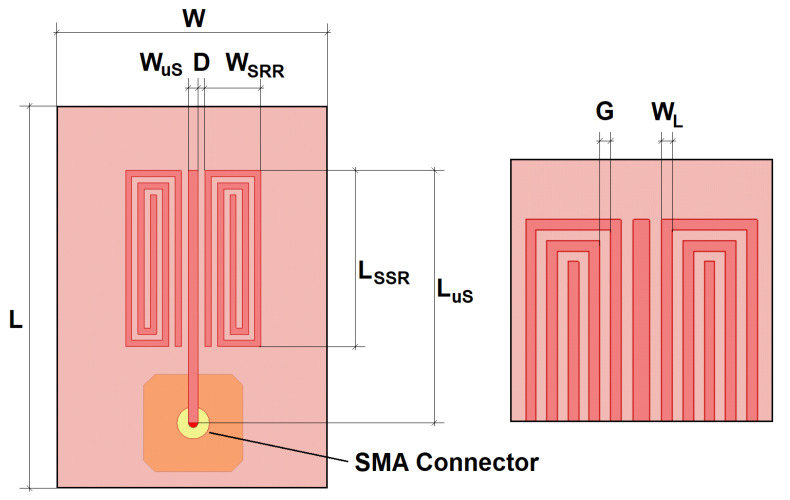
Sketch of the B-type SRR-based sensor. The inset shows a detail of the microstrip open termination.

Figure 3 sketches the B-type sensor. Each resonator has a length $L_{\mathrm{SRR}}$ of 23 mm and a width $W_{\mathrm{SRR}}$ of 7.2 mm. Both line widths $W_{L}$ and gaps *G* are 0.8 mm. The distance between the resonators and the microstrip line, *D*, is 0.93 mm. The microstrip line is 1.22 mm wide, which corresponds to a $Z_{0}=50\;\Omega$ characteristic impedance. The resulting substrate dimensions, L×W, are $35\times 30\;{\mathrm{mm}}^{2}$.

Due to the substrate porosity and roughness, the fluid to be measured may contaminate the sensor, which would make it unfit for future measurements. Furthermore, the presence of conductive compounds in the sample may short-circuit the resonators. Because of this, all manufactured sensors are protected with a 25 μm-thick Kapton layer (Figure 2b,c). This protective layer has a relative permittivity $\varepsilon_{r}=3.4$ and a loss tangent tan(δ)=0.0018, which must be taken into account in the resonator design.

**Figure 4:**
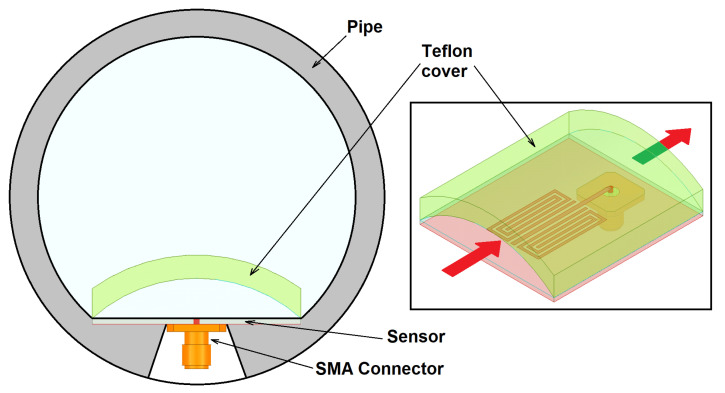
Integration of the sensor inside of a pipe (gray). A Teflon cover is placed above the sensor for ensuring an axial flow near the SRR resonator. Only part of the fluid (blue) contributes to the measurement. For obtaining a relevant sample, a homogeneous flow must be achieved. The inset shows a 3D view of the cover and the sensor.

The B-type sensor can be easily integrated inside a pipe for the real-time monitoring of a fluid flow. Figure 4 sketches this scenario. A Teflon cover must be placed above the sensor for ensuring a continuous axial flow. This cover was also considered in the full-wave simulations.

**Figure 5:**
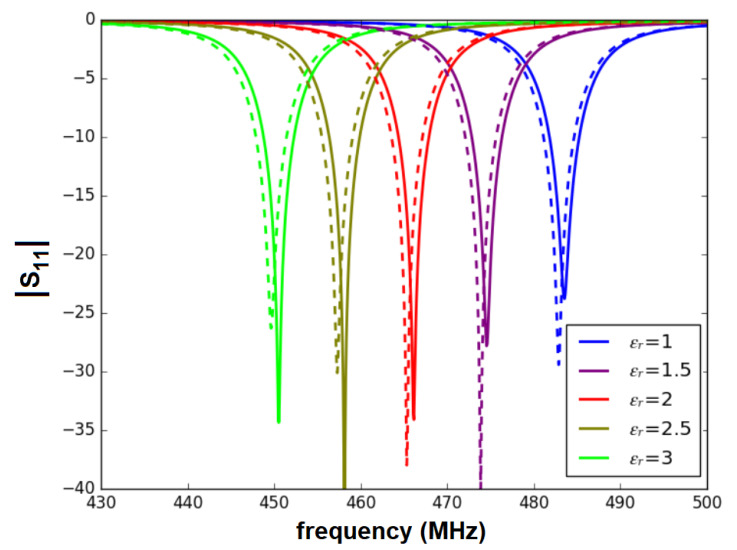
Simulated $|S_{11}|$ for the uncovered (solid) and covered (dashed) sensor. Fluids of permittivities between 1 and 3 are considered.

All the full-wave simulations were performed with Ansys HFSS v19 ©. Figure 5 shows the calculated $|S_{11}|$ when considering fluids of a relative permittivity from one to three. As expected, the resonant frequency $f_{0}$ shifts to lower frequencies when increasing the fluid permittivity $\varepsilon_{{}_{\mathrm{LUT}}}$. As expected, the Teflon cover creates a slight shift of $f_{0}$ that must be taken into account.

Only a one-dimension measurement, related to the complex magnitude $\Gamma =S_{11}$, is required for determining the resonant frequency. In Section 4, a cost-effective 1D interrogator for the SRR sensor is introduced. It is an interesting alternative to a 2D S-parameter determination using a one-port VNA, especially for multiple-sensors systems.

Next, we demonstrate that the resonant frequency measurement of SRR sensors allows the determination of different sample parameters.

## 3. Crude Properties Estimation

All measurements shown in this section have been undertaken with a Keysight N9914A Fieldfox VNA. The module of the S${}_{11}$ parameter of the resonator was obtained in order to monitor its resonant frequency $f_{0}$. The mixtures were prepared by taking into account the mass fraction of each of its components. Using a magnetic mixer ensures sample homogeneity. All measurements discussed in this section were taken under normal ambient temperature and pressure.

### 3.1. Discrimination between Different Crude Samples

**Figure 6:**
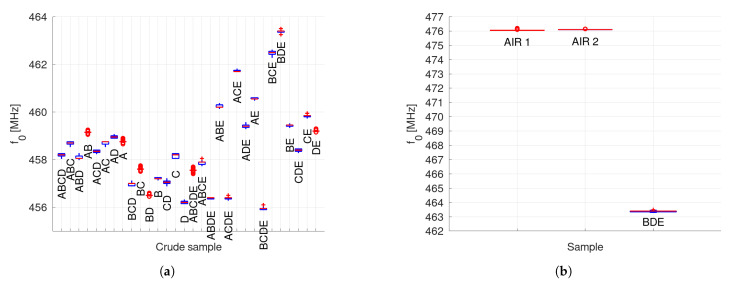
Resonant frequencies $f_{0}$ for different mixtures (**a**). Air was measured before and after the crudes (**b**). Since the same resonant frequency was obtained, the SRR sensor survived all the measurements between without any damage or substrate contamination. BDE sample is also shown.

During the experiments, five different crude samples, labeled as A, B, C, D, and E were used. Each of them were obtained from different wells around the world. Due to the differences in their composition, the permittivity is different and, therefore, also the measured resonant frequency (Figure 6a). As expected, when mixing crudes, newer permittivities are obtained. All possible combinations (binary, ternary, quaternary, and quinary) were also characterized. All mixtures have an equal mass fraction for each of their compounds. Figure 6a is a box plot that summarizes 100 measurements per sample of the immersed sensor $f_{0}$. Since $f_{0,air}$ (Figure 6b) keeps the same before (AIR 1) and after (AIR 2) measuring the crudes, it is obvious that no damage or contamination has been produced.

### 3.2. Sulfur Concentration Estimation

**Figure 7:**
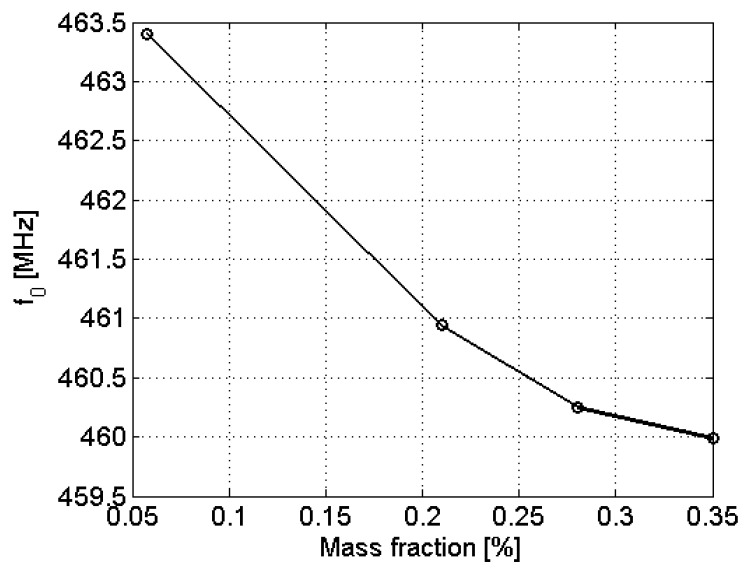
Evolution of the resonant frequency $f_{0}$ with the mass fraction of sulfur.

Next, a group of samples with different sulfur concentrations were prepared from the same crude. Figure 7 shows the resonant frequency over the mass fraction. The best sensitivity is achieved for mass fractions of sulfur between 0.05% and 0.36%. Actual limits of measurable concentrations are related to the achieved frequency resolution. For concentrations below 0.05%, the effect of sulfur in the permittivity of the sample is negligible.

### 3.3. Aromatic Hydrocarbons Concentration Estimation

**Figure 8:**
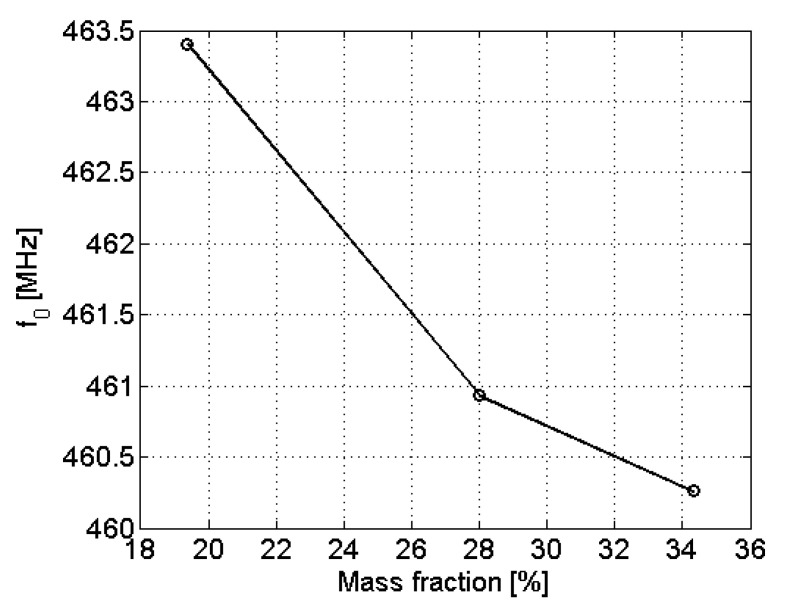
Evolution of the resonant frequency $f_{0}$ with the mass fraction of aromatic hydrocarbons.

The same crude sample was used for evaluating the ability to detect aromatic hydrocarbons concentrations with SRR sensors. Because of this, a low concentration leads to the same resonant frequency ($f_{0}=$ 463.4 MHz), which corresponds to the specific crude sample. The highest sensitivity is achieved for mass fractions between 20% and 36% (Figure 8).

### 3.4. Salt-Water Concentration

**Figure 9:**
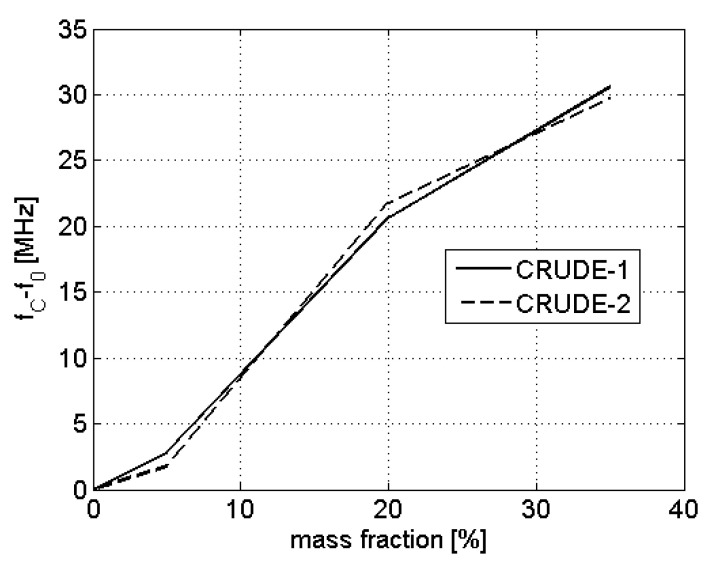
Evolution of the resonant frequency difference $f_{0,C}-f_{0}$ with the mass fraction of salt-water for mixtures created from two different samples: CRUDE-1 (solid) and CRUDE-2 (dashed).

Finally, we prove that the sensor can estimate the amount of salt-water inside the crude fluid. Two different crude samples (CRUDE-1 and CRUDE-2, with resonant frequencies of $f_{0,C}=$ 459.2 MHz) were used for generating this series of mixtures. A sample of Mediterranean water (resonant frequency of 282.6 MHz) was used as the salt-water. For each crude, four different mixtures of 0%, 5%, 20%, and 35% salt-water mass fractions were prepared. Figure 9 shows the frequency deviation in MHz from the 0% salt-water concentration. As can be seen, the frequency deviation is dependent on the salt-water concentration and independent of the CRUDE sample used in the mixture preparation.

Since many crude properties can be obtained from the SRR-resonant frequency, we have developed a cost-affordable sensor interrogator as an alternative to the $S_{11}$ measurement using VNAs. Our proposed system can be remotely operated and does not require trained personnel, since it is fully autonomous after being installed.

## 4. SRR Sensor Interrogator

**Figure 10:**
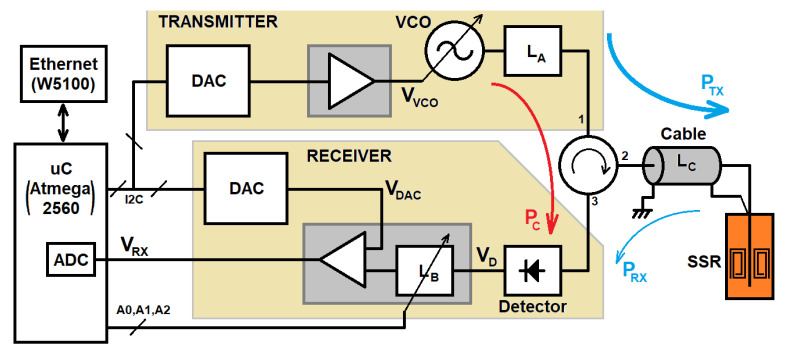
Sketch of the system.

For a real-time permittivity measurement, the following topology is proposed (Figure 10). A continuous-wave voltage-controlled source injects power $P_{\mathrm{TX}}$ into the sensor through the circulator. The reflected power $P_{\mathrm{RX}}$ in the SRR is sent back to the detector through the circulator. An Atmega2560 microcontroller sets the VCO frequency via the transmitter digital to the analog converter (DAC). The receiver output voltage $V_{\mathrm{RX}}$ is then digitized by using the microcontroller ADC. All the measurement parameters (frequency, integration time, and scanning bandwidth, etc.) are configured via HTTP commands and sent through a standard TCP/IP Ethernet connection. A low-cost Arduino MEGA2560 and a W5100-based Ethernet shield are used for implementing the control electronics.

A Mini-Circuits ZX95-625+ is used as the VCO. A DPVCC45A circulator working between 410 MHz and 500 MHz is used for connecting the sensor to both the transmitters and receivers. The power detector is a Mini-Circuits ZX47-60LN+.

Next, both the transmitter and the receiver design is highlighted.

### 4.1. Transmitter

The VCO is commanded by an I^2^C 12-bit MCP4725 DAC. The DAC outputs voltage varies from 0 to 5 V. To maximize the frequency resolution, the $V_{\mathrm{OSC}}$ voltage range is tailored according to its response curve for the frequency range between 421.13 MHz and 506.5 MHz, which corresponds to a voltage level from 5.8 V to 9 V. This is achieved via offset and amplitude compensation with a TL081 set in a non-inverting op-amp configuration. Therefore, $2^{12}=4096$ frequency steps imply a maximum resolution of ≈20.85 kHz.

The VCO generates a power level of 6 dBm for frequencies from 400 MHz to 520 MHz. Due to the measured circulator isolation between ports one and three ($max(|S_{31}|)=-24.6$ dB), an attenuator of $L_{A}=16.3$ dB is placed at the VCO output. The VCO power coupled to the detector $P_{C}$ is then reduced to −34.3 dBm. The maximum power level in the detector is 6 dBm − 16.3 dB = −10.3 dBm.

### 4.2. Receiver

**Figure 11:**
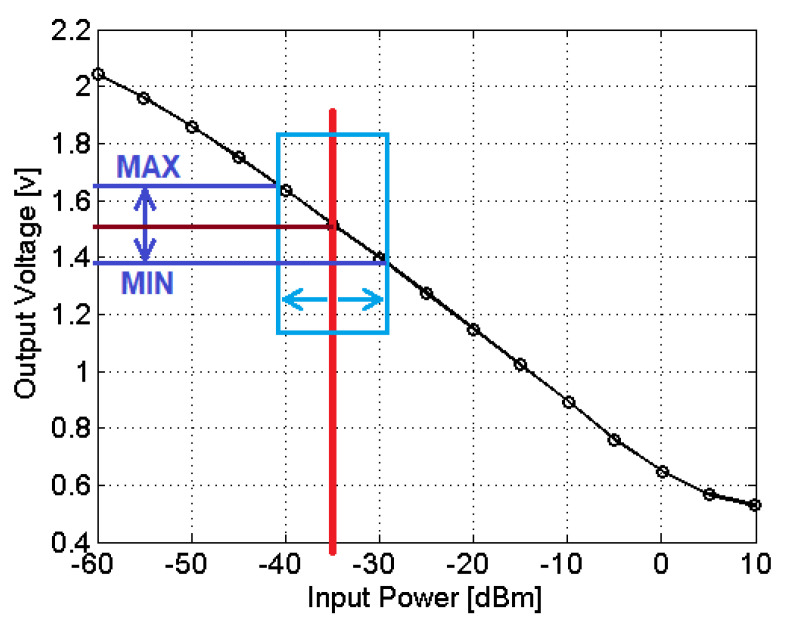
Response curve of the Mini-Circuits ZX95-625+ power detector [28].

The power detector response curve is shown in Figure 11 [28]. As can be seen, the output voltage $V_{D}$ is inversely proportional to the input power in dBm. The red line shows the power level $P_{C}$. Depending on the phase between the VCO-coupled power and the received signal, the power level in the detector can increase or decrease (blue). Both the cable and the sensor affect the receiver signal amplitude and phase. In this design, the cable impact is assumed to be constant and can be compensated by proper signal conditioning after the detector.

**Figure 12:**
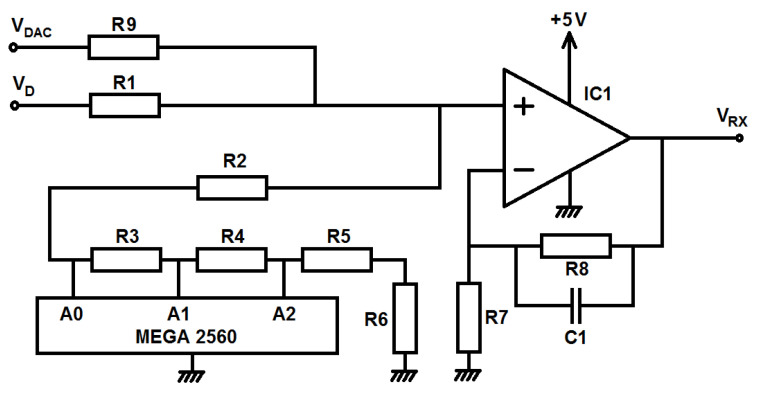
Simplified schematic of the receiver signal conditioner.

The receiver offset and amplitude compensation is performed with a variable attenuator and a TL081 non-inverting op-amp configuration. The schematic of this topology is shown in Figure 12. An MCP4725 DAC synthesizes the voltage $V_{\mathrm{DAC}}$ for the offset compensation. A resistive divider attenuates both $V_{\mathrm{DAC}}$ and the signal-detected $V_{D}$. A set of resistors, R2, R3, R4, R5, and R6, has been added to conform a variable resistor controlled by the three ports A0, A1, and A2. Each port can be configured as a low-state output or a high, which corresponds to low- and high-impedance status. This allows four different attenuation $L_{B}$ values. Since the amplifier has a constant gain G= 151, a lower attenuation can be used for compensating higher cable losses.

The capacitor $C_{1}$ limits the measurement speed and filters the high-frequency noise ($f_{C}=$ 48.22 Hz). For flattening the microcontroller consumption, the Atmega2560 ADC is kept measuring continuously, which drastically reduces the measurement noise. All the samples taken during the measurement time $t_{m}$ are averaged. $t_{m}$ can be set from 300 ms to 30 s. Higher values can further improve the cable loss compensation. When using a 25 dB attenuator for simulating cable losses (total loss of 50 dB) and $t_{m}=$ 400 ms, the sensor resonance can be still detected. With proper cable selection (i.e., Times Microwave HP1200 or Commscope AVA7-50, with losses of 3.4 dB/km and 1.53 dB/km, respectively) distances between the interrogator and the sensor above 1 km are feasible.

**Figure 13:**
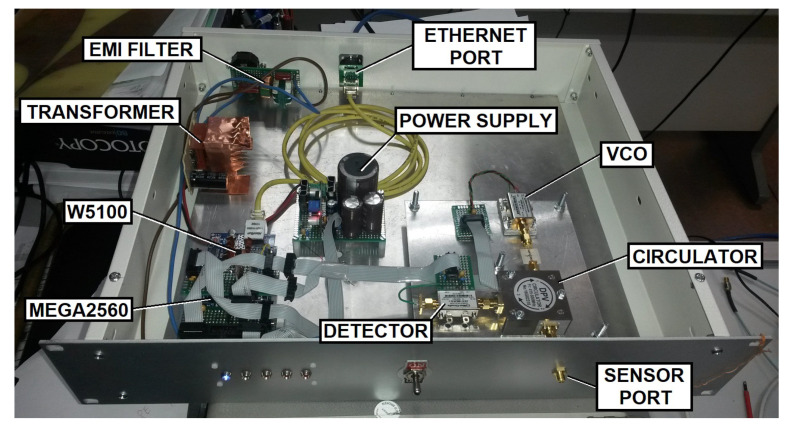
Manufactured SRR sensor interrogator.

Figure 13 shows the prototype. It is integrated into a 19-inch universal case. The radio frequency elements are integrated into a dedicated aluminum block as an independent sub-assembly. This metal block is designed to have a large heat capacity. It allows thermal stability for both the VCO and the detector without active temperature control through either thermoelectric cooler or fans. The system stability is demonstrated in Section 5. The front panel has a single SMA female port on which the sensor is connected.

All the required operations are performed remotely via SCPI-compatible HTTP commands. It can work as a stand-alone device or as a part of a multi-sensor system. Both the SRR-resonant frequency and the full measurement vector can be obtained through HTTP commands. When measuring the SRR-resonant frequency in a stand-alone mode, only one-dimension data are extracted from the crude sample, so it is not possible to monitor the change of more than one property at the same time.

## 5. High Pressure and High Temperature Measurements

**Figure 14:**
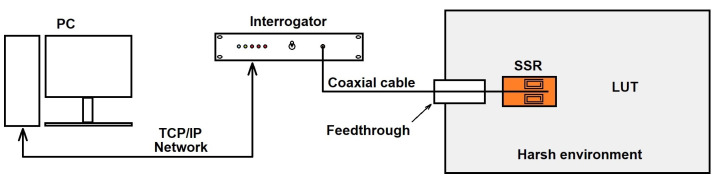
Sketch of the high-pressure and high-temperature measurement setup.

The system was tested in a laboratory environment. The sensor is integrated into a bottle where the high pressures (up to 970 bar) and the high temperatures (up to 200 °C) are generated when filling with the liquid under test (LUT). The LUT constantly recirculates to avoid stratification. The measurement setup is sketched in Figure 14. Only the SRR sensor and a coaxial segment work in a high-pressure and high-temperature environment. The interrogator, the personal computer (PC), and the TCP/IP network run at a normal ambient pressure and temperature.

**Figure 15:**
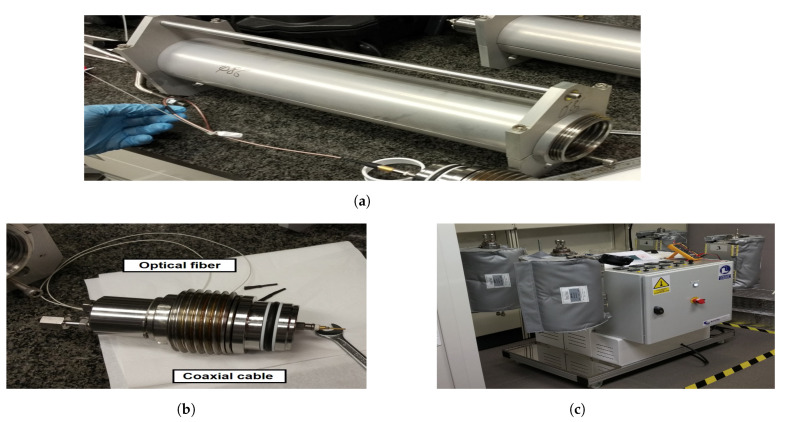
High-pressure and high-temperature measurement setup: high-pressure bottle (**a**), feed-through (**b**), and bottle holder and heater (**c**).

Figure 15 shows the experimental setup. The SRR sensor is fitted inside the high-pressure bottle (Figure 15a). The feed-through (Figure 15b) allows the radio frequency connection between the high-pressure and the low-pressure coaxial cable sections. It can also accommodate several optic fibers for multi-sensor characterization systems. The bottle is fixed to a holder that controls the experiment temperature (Figure 15c).

### 5.1. System Stability

**Figure 16:**
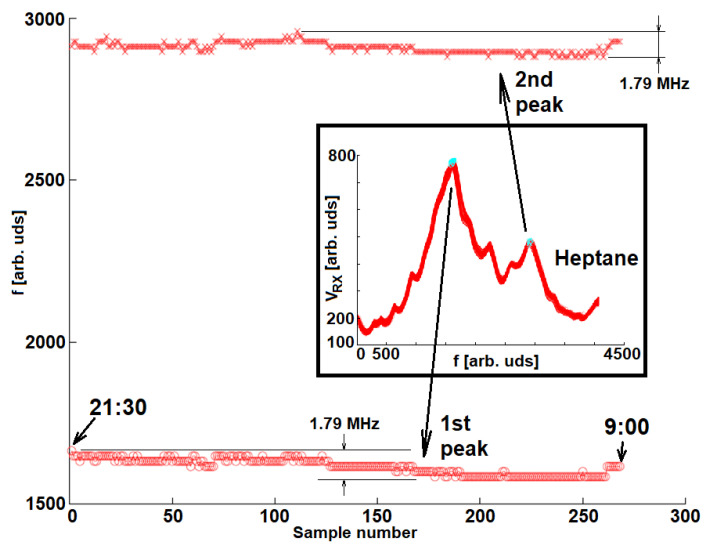
Frequency of the first and second $V_{RX}$ peaks along time. No drifts can be appreciated when no parameters are changed in the high-pressure bottle. The inset shows all $V_{RX}$ sweeps overlapped.

Several crude samples and mixtures were characterized in different measurement rounds. The interrogator was kept on and measuring during the days that the experimental work was undertook, which allowed us to test the stability of the system after some time. Figure 16 shows the evolution of the two main peaks of $V_{RX}$ when measuring heptane continuously during 11 h 30 min. The frequency resolution was 358.1 kHz so the frequency determination was inside the $\bar{f_{0,H}}\pm 895.2$ kHz interval, $\bar{f_{0,H}}$ being the mean of all resonant frequencies.

### 5.2. VNA and Interrogator Comparison

**Figure 17:**
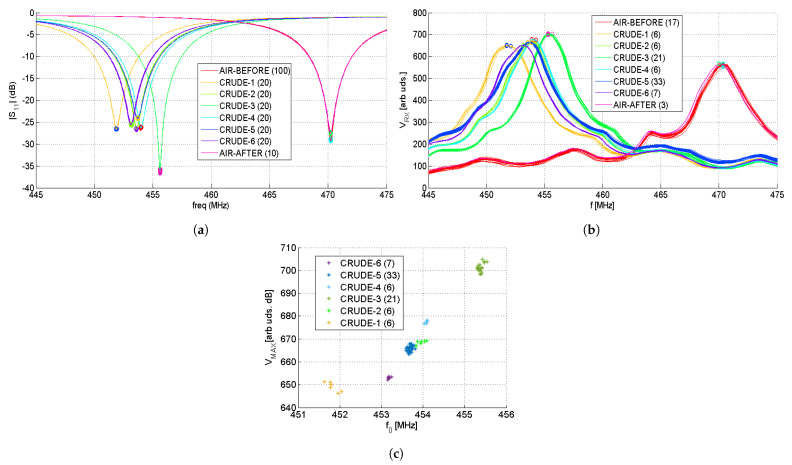
Measurements of six crude samples using the same SRR sensor, the VNA (**a**), and the Interrogator (**b**). The inset shows the number of measurements performed for each sample. The 2D plot of V${}_{\mathrm{MAX}}$ and f${}_{0}$ of each measurement (**c**). The air measurements are not shown.

Six crude samples were measured with the same SRR sensor driven by using both the Keysight N9914B VNA and the developed interrogator in order to qualitatively compare their discrimination capabilities. They are plotted in Figure 17 in the frequency range from 445 MHz to 475 MHz. Figure 17a shows the module of the $S_{11}$ parameter measured with the VNA in dB. Figure 17b shows the measured voltage $V_{RX}$ (see Figure 10 and Figure 12).

The resonant frequency can be obtained by finding the minimum in the module of the $S_{11}$ curve or the peak V${}_{\mathrm{MAX}}$ in the $V_{RX}$ curve. The resonant frequencies $f_{0}$ and the achieved standard deviations σ are summarized in Table 1. The VNA achieves a standard deviation that is one order of magnitude below the one achieved by our solution. For this experiment, the interrogator was configured with a measurement time of $t_{m}=400$ ms. Smaller σ are achievable when increasing $t_{m}$. For the achieved value of σ, it is not obvious that a crude differentiation can be performed when considering only f${}_{0}$. In our application, it was possible to do it thanks to the SVM that takes into account all the measured points. If our interrogator is used as a stand-alone system, a rudimentary classifier can be implemented in the microcontroller. Besides f${}_{0}$, other parameters, such as the peak amplitude V${}_{\mathrm{MAX}}$ and the peak width, can be considered. As an example, Figure 17c shows f${}_{0}$ and V${}_{\mathrm{MAX}}$ for each measurement. Although crude samples two, four, and five have a close f${}_{0}$, the classification is still possible in the 2D plane.

As it can be appreciated, there is an agreement between the VNA and the interrogator measurements. Air was measured before and after all crude characterization. Since there is an agreement between both air measurements, we can conclude that the sensor is not contaminated or degraded and both the VNA and the implemented interrogator remain frequency-stable during the whole experiment, as expected after the results shown in Figure 16. Both systems were able to discriminate between the different samples.

## 6. Conclusions

In this document, an SRR sensor-based system is proposed for the real-time monitoring of crude properties for the petroleum industry. The sensor can be integrated into the well’s pipes and can work at high-pressure and high-temperature conditions.

Experimental work has been carried out for demonstrating that the system can discriminate between different crude samples. It is also able to determine the sulfur, the aromatic hydrocarbons, and the salt-water concentrations. It is obtained by detecting permittivity changes by measuring the corresponding SRR-resonant frequency.

A cost-affordable sensor interrogator has been developed. It is an autonomous system that can be remotely operated via HTTP commands sent through a TCP/IP network.

The whole system has been validated at high-pressure and high-temperature working conditions. The system stability has been tested through several weeks of continuous measurements. Nevertheless, further work is required in order to test our solution in a more realistic environment, since the effects of high pressure and high temperature on coaxial connectors could degrade the performance of the whole system. Furthermore, our solution should be compared with other state-of-the-art alternatives in on-site tests.

## Figures and Tables

**Table 1:** List of the measured resonant frequencies and standard deviation $\sigma =|f_{0}-\bar{f_{0}}{|}^{2}/N$ for the same sensor driven by the VNA and the implemented interrogator for different crude samples.

Sample	VNA $\bar{f_{0}}$ [MHz]	Interrogator $\bar{f_{0}}$ [MHz]	VNA σ	Interrogator σ
Air (before)	470.22	470.35	0.025	0.000
Air (after)	470.22	470.11	0.024	0.207
CRUDE-1	451.88	451.85	0.038	0.185
CRUDE-2	453.60	453.88	0.016	0.000
CRUDE-3	455.61	455.33	0.022	0.078
CRUDE-4	453.97	454.06	0.038	0.196
CRUDE-5	453.71	453.63	0.026	0.167
CRUDE-6	453.14	453.16	0.037	0.000

## Data Availability

Not applicable.
